# Magnetic Resonance-Based Analytical Tools to Study Polyvinylpyrrolidone–Hydroxyapatite Composites

**DOI:** 10.3390/polym15224445

**Published:** 2023-11-17

**Authors:** Alina Petrova, Georgy Mamin, Oleg Gnezdilov, Inna Fadeeva, Olga Antonova, Anna Forysenkova, Iulian V. Antoniac, Julietta V. Rau, Marat Gafurov

**Affiliations:** 1 Institute of Physics, Kazan Federal University, Kremlyovskaya St.18, 420008 Kazan, Russia; petrovaalinakfu@gmail.com (A.P.); goi@yandex.ru (O.G.); 2 A.A. Baikov Institute of Metallurgy and Material Science, Russian Academy of Sciences, Leninsky Avenue 49, 119334 Moscow, Russia; fadeeva_inna@mail.ru (I.F.); aforysenkova@gmail.com (A.F.); 3 Faculty of Material Science and Engineering, National University of Science and Technology Politehnica Bucharest, 313 Splaiul Independentei Street, District 6, 060042 Bucharest, Romania; antoniac.iulian@gmail.com; 4 Academy of Romanian Scientists, 54 Splaiul Independentei Street, District 5, 050094 Bucharest, Romania; 5 Istituto di Struttura della Materia, Consiglio Nazionale delle Ricerche (ISM-CNR), Via del Fosso del Cavaliere, 100, 00133 Rome, Italy; giulietta.rau@ism.cnr.it; 6 Department of Analytical, Physical and Colloid Chemistry, I.M. Sechenov First Moscow State Medical University, Trubetskaya Str., Build. 8/2, 119048 Moscow, Russia

**Keywords:** polyvinylpyrrolidone, hydroxyapatite, composites, NMR, EPR

## Abstract

The synthesis of biocompatible and bioresorbable composite materials, such as a “polymer matrix-mineral constituent,” stimulating the natural growth of living tissues and the restoration of damaged parts of the body, is one of the challenging problems in regenerative medicine and materials science. Composite films of bioresorbable polymer of polyvinylpyrrolidone (PVP) and hydroxyapatite (HA) were obtained. HA was synthesized in situ in the polymer solution. We applied electron paramagnetic resonance (EPR) and nuclear magnetic resonance (NMR) approaches to study the composite films’ properties. The application of EPR in two frequency ranges allowed us to derive spectroscopic parameters of the nitrogen-based light and radiation-induced paramagnetic centers in HA, PVP and PVP-HA with high accuracy. It was shown that PVP did not significantly affect the EPR spectral and relaxation parameters of the radiation-induced paramagnetic centers in HA, while light-induced centers were detected only in PVP. Magic angle spinning (MAS) ^1^H NMR showed the presence of two signals at 4.7 ppm and −2.15 ppm, attributed to “free” water and hydroxyl groups, while the single line was attributed to ^31^P. NMR relaxation measurements for ^1^H and ^31^P showed that the relaxation decays were multicomponent processes that can be described by three components of the transverse relaxation times. The obtained results demonstrated that the applied magnetic resonance methods can be used for the quality control of PVP-HA composites and, potentially, for the development of analytical tools to follow the processes of sample treatment, resorption, and degradation.

## 1. Introduction

**Figure 1:**
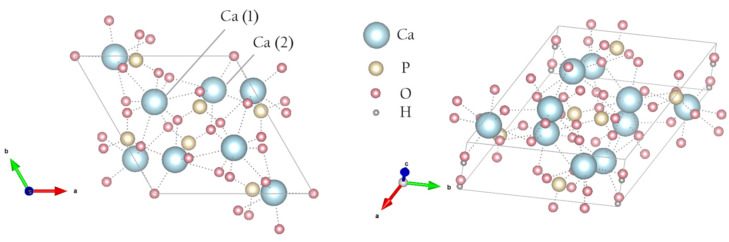
Structure of HA.

Calcium phosphates (CaPs) are the main inorganic component of bone and dental tissues [1,2,3,4,5]. Materials based on CaPs—powders, ceramics, cements, coatings, and composites—are widely used in various applications, especially in medicine for the replacement and restoration of damaged bone tissues. The most widespread representative of CaPs is hydroxyapatite (HA, Ca_10_(PO_4_)_6_(OH)_2_, Figure 1), which is present in bone tissues in the form of nanocrystals. HA is known to be biocompatible, non-toxic, and the most important mineral that constitutes bones and teeth [6].

**Figure 2:**
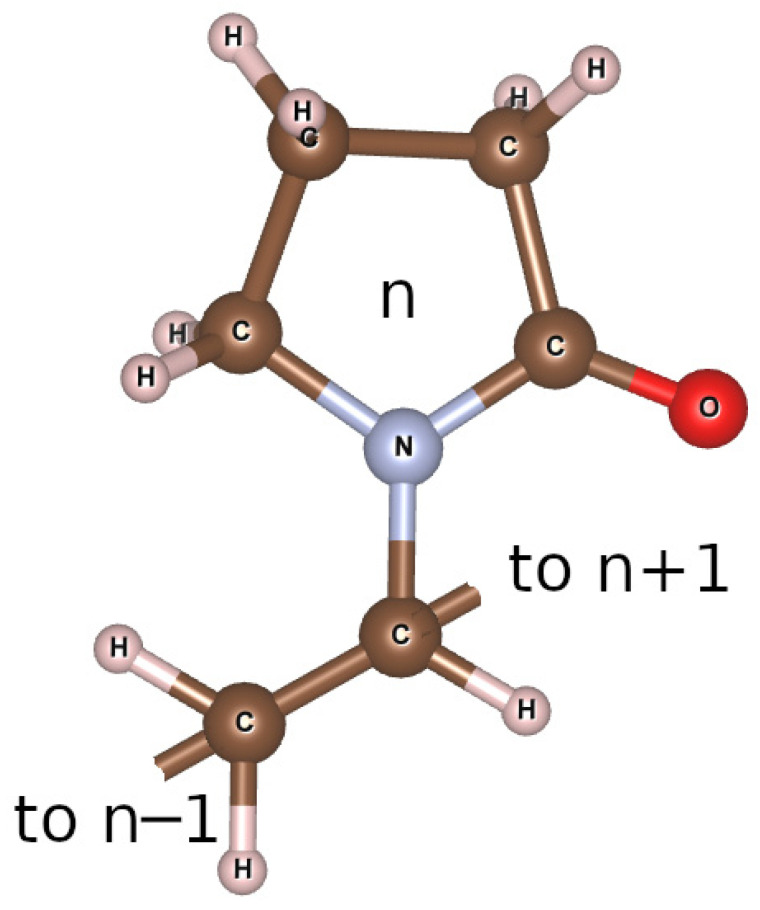
Schematic structure of PVP.

It is known that the synthesis of calcium phosphates in the presence of polymers (collagen, gelatin, starch, chitosan, etc.) leads to the formation of CaP nanocrystals with controlled size, morphology, and improved mechanical properties [7,8,9,10]. Currently, 3D-printing technologies are rapidly developing in medicine, in particular for printing bone implants. CaP frames printed using a 3D printer for biomedical applications have good mechanical strength [11]. In the standard 3D technology for creating such objects, composites made of a biodegradable polymer and a calcium-containing filler are applied for layer-by-layer deposition of the material [12]. Polyvinylpyrrolidone (PVP, Figure 2) is widely employed as a synthetic polymer for various biomedical purposes in material engineering due to its diverse properties, including solubility in water and in a broad range of liquid media. PVP also has both hydrophilic and hydrophobic functional groups, due to which it interacts with various solvents (easily soluble in cold water and also soluble in many organic solvents, including alcohols; some chlorinated compounds such as chloroform, methylene chloride and ethylene dichloride; nitroparaffins; and amines [13,14,15,16]). Thanks to its biocompatibility, absence of toxicity and high capacity to form interpolymer complexes, PVP is widely used for designing materials for different applications, such as biomaterials for medical uses.

Bone cements are often used to fix artificial prostheses to the human skeletal system. These materials provide immediate fixation of the implant and ensure a better distribution of body load between the prosthesis and bone. Morejón et al. [17] studied polymer particles with particle size distribution and molecular weight, allowing them to develop bone cement according to the international standards. Needle-shaped HA crystals were obtained in an aqueous solution of PVP via precipitation from solutions of calcium nitrate and orthophosphoric acid at 60 °C. PVP was used to regulate the nucleation and growth of HA crystals. Based on the results obtained from X-ray diffraction and high-resolution transmission electron microscopy measurements, it was concluded that the addition of PVP promotes the growth of HA nanocrystals [18,19]. It was shown that the number of HA crystals formed on the polymer surface depends on the number of negative charges on this surface and the pH of the reaction medium. It was established that to obtain a controlled morphology, the temperature and duration of the reaction are of primary importance [20]. 

Polymer—CaPs nanocrystal composites can be obtained in situ, without mixing the components, similar to the process happening in the body during bone remodeling. In ref. [21], such a composite was obtained via biomimetic deposition of HA on a PVP matrix. A colorimetric test to assess the metabolic activity of cells (MTT-test) showed that the composites were biocompatible and could be used to fill bone defects. Guesmi Y. et al. [22] investigated the material obtained by grafting PVP onto the surface of HA microcrystals, suitable for bone tissue engineering. Based on the results of the bioactivity study of the composites, it was concluded that the PVP seeded on their surface cells had a higher viability compared to those cultivated on the HA crystals.

Obviously, the properties of polymer-CaP composites depend on the chemical interaction of the components. It is supposed that the inorganic particles interact with the organic component by establishing the inter- and intramolecular hydrogen bonds and via the ion–dipole forces, formed, for example, between the calcium ions of CaPs and the functional groups of polymers [23,24,25,26]. However, the interaction of PVP with calcium phosphate microcrystals has not yet been sufficiently studied. 

A lot of work has been conducted on the development and application of various characterization methods that can provide details about calcium phosphate-based biomaterials at different scales, such as X-ray diffraction, infrared and electron microscopy, rheological analysis, water content determination, etc. [8]. In the present work, PVP-HA composites were investigated using electron paramagnetic resonance (EPR) and nuclear magnetic resonance (NMR) techniques. Both of these analytical methods are non-invasive, widely applied in material science, polymer research and for biomedical purposes, but very rarely exploited for polymer-CaPs studies. 

Pure HA and PVP are EPR silent. Therefore, due to its inherent high sensitivity, EPR can be used to check for the presence of various paramagnetic impurities, like metals and metal complexes, or defects in the polymer chains both in the starting materials for synthesis and in the resulting products [27,28,29,30,31,32,33,34]. Stable and unstable defects created by light or ionizing irradiation (X-ray irradiation, for example) can be exploited not only for detecting the presence and concentration of impurities but also as paramagnetic probes to study the local environment, material structure and changes in the local lattice [27]. Several stable radiation-induced anions were detected and investigated using EPR in synthetic and biogenic CaPs, like carbonates, nitrogen and oxygen-containing species [30,32,33,34]. It was shown, for example, that the spectra of nitrogen-containing stable radicals depend on the type of CaP and, therefore, EPR may be used to follow the processes of calcium phosphates growth [28,29]. In HA, the mentioned anions can substitute both hydroxyl (OH) and phosphate (PO_4_) groups (see Figure 1), as well as occupy interstitial sites. The localization of anions depends on the nature of biogenic materials, the method used for the synthesis of artificial compounds, co-doping effects, etc. Advanced EPR techniques, together with theoretical calculations, often allow one to define the location of these anions in the HA structure, and to propose the most probable charge compensation schemes, in the case of non-isovalent substitutions [27,29].

EPR techniques are used to investigate intentional or non-intentional HA cation doping with Mn^2+^, Cu^2+^, etc. Two distinct sites, Ca(1) and Ca(2), are distinguished in the HA lattice (Figure 1), and EPR data allow one to confirm or refute assumptions about the introduction of the dopants into the HA structure, preferential substitution or re-distribution of dopants between the calcium sites [27]. Even in the case of non-paramagnetic impurities, such as magnesium and aluminum, for example, it was shown that the EPR of anionic impurities is sensitive to the lattice distortion caused by these cations [29].

Due to the chemical complexity of polymer-CaPs composites, hyperfine EPR and NMR can be engaged, taking into account that different nuclei, such as ^31^P (with the nuclear spin of I = 1/2), ^1^H (I = 1/2), ^13^C (I = 1/2), ^14^N (I = 1) and even ^43^Ca, can act as nuclear spectroscopic probes. Since HA is practically insoluble in liquid media, magic angle spinning (MAS) NMR techniques are used to investigate materials containing hydroxyapatite [35,36,37,38,39,40,41,42]. Thanks to the advancements in instrumentation, pulse sequence development and data interpretation (through combined experimental–computational approaches) made by the NMR community since the beginning of 2000s, materials scientists have been able to extend the limits of NMR research in order to obtain a deeper knowledge of CaPs biomaterials [36]. NMR studies of native bone and dental tissues have allowed us to measure the size of the amorphous calcium phosphate layer at the surface of apatite bone crystallites, to detect minor organic species (like nucleic acids) and to measure the distance and binding geometry of small molecular ions at the surface of mineral particles, like citrate. Furthermore, significant information about inorganic and organic components, as well as the exclusion of previously suggested models regarding the bone structure and its formation, has been obtained by comparing the NMR signatures of natural materials with those of synthetically derived models, including in vitro-cultured tissues [36]. The achievements in this field give hope that in the near future, novel approaches can be implemented into clinical magnetic resonance imaging (MRI) for the evaluation of skeleton quality, 3D and 4D synthetic tissue constructs and the diagnosis of bone diseases in order to provide additional biomarkers for the assessment of bone microarchitecture, etc. [37].

The effect of PVP on the morphology and size of nanocomposites was studied using NMR in refs. [17,40]. In several papers from our group, we demonstrated the feasibility of combined EPR/NMR studies to investigate PVP-HA-sodium alginate composites, including the influence of divalent metals on the cross-linking of the polymer and HA [35,41]. However, to the best of our knowledge, no comprehensive analysis of synthesized in situ PVP-HA composites using the EPR and NMR techniques is present in literature.

## 2. Materials and Methods

### 2.1. Synthesis of Composite Materials Based on Polyvinylpyrrolidone with Hydroxyapatite

Solutions of Ca(NO_3_)_2_ × 4H_2_O (chemical grade, PanReacAppliChem, Barcelona, Spain), (NH_4_)_2_HPO_4_ (chemical grade, Chimmed, Moscow, Russia) and (PVP) Mw = 12 kDa (Boai NKY Pharmaceuticals Ltd., Tianjin, China) were used for the in situ synthesis of HA powders in the PVP solution. Precipitation of calcium phosphates was performed at room temperature (20–25 °C) at pH of ~11.5, in accordance with Equation (1) [41]:

10Ca^2+^ + 6HPO_4_^2−^ + 8NH_3_ H_2_O → Ca_10_(PO_4_)_6_(OH)_2_ + 8NH_4_^+^ + 6H_2_O (1)

The in situ synthesis of PVP-HA composites was carried out according to the procedure described in ref. [40]. Briefly, an aqueous PVP solution was prepared by dissolving 14.35 g of PVP in 200 mL of distilled water via stirring, in order to obtain 5.52 wt% concentration. After the formation of a homogeneous water–polymer mixture, 20 mL of 0.1 mol/L calcium nitrate solution was added. To regulate the reaction acidity, 20 mL of 25% NH_4_OH aqueous solution was added as well. Then, 20 mL of 0.06 mol/L diammonium phosphate solution was added drop by drop while stirring at 500 rpm. To remove the by-products of the HA formation (reaction (1)) (i.e., nitrate ions) from the composite, it was washed using dialysis.

The product of reaction (1) was examined with X-ray diffraction (XRD) and Fourier transform infrared spectroscopy (FTIR). The XRD analysis was carried out by using the DRONE 3M diffractometer (S.-Petersburg, Russia) with Cu Kα radiation (λ = 0.154 nm). The FTIR spectra were obtained with the NicoletAvatar-330 (ThermoScientific, Waltham, MA, USA) spectrometer in the range of 400–4000 cm^−1^ on the samples mixed with potassium bromide. The annealing of samples before XRD and FTIR was performed for 1 h at 400 and 900 °C. The details of the samples characterization were reported in ref. [41].

From XRD, it was found that the resulting composites are X-ray amorphous. As a result of heat treatment at 400 °C, an apatite structure was formed, and after heat treatment at 900 °C, only crystallized HA without admixtures of other phases was detected.

FTIR measurements showed that in the sample without heat treatment, despite a partial overlap of PVP and phosphate group bands, it is still possible to distinguish the bands related to PVP. After the comparison of FTIR spectra with the data reported in ref. [41], it was found that the most intense bands at 1660 cm^−1^ correspond to the valence vibrations of the carbonyl group (C=O). In the spectra related to HA, the oscillations for the PO_4_^3−^ group were pronounced (triplets of valence oscillations at 1090, 1053 and 965 cm^−1^ and at 632, 572 and 472 cm^−1^). The intense oscillation band of the OH-group at 3570 cm^−1^, which is present even in the sample without heat treatment, may indicate the beginning of the formation of the HA structure. With an increase in the annealing temperature, the intensity of this band significantly increased, allowing it to be associated with the crystalline hydroxyapatite formation. The intensity and resolution of the bands corresponding to the PO_4_^3−^ group also increased. It should be noted that the PVP bands practically disappear after heat treatment at 400 °C, and completely disappear after annealing at 900 °C. 

Scanning electron microscopy (SEM) measurements were performed using a Tescan VEGA3 (Kohoutovice, Czech Republic). Samples were coated with a thin layer of gold for the SEM examinations. The SEM images were acquired using secondary electron (SE) and backscattered electron (BSE) imaging modalities. The results of SEM investigations are presented in Section 3.1.

### 2.2. EPR

The EPR measurements were performed in two microwave ranges, X-band (with the microwave frequency ν_MW_ = 9 GHz) and W-band (ν_MW_ = 94 GHz), by exploiting the Bruker Elexsys E580/E680 spectrometer (Karlsruhe, Germany) in conventional (cw) and pulsed modes. Pulse methods are able to provide much more information compared to that obtained using conventional EPR [43]. In the pulsed mode, the Hahn sequence was applied to detect electron spin echo (ESE): π/2–τ–π–τ–; here, the duration of π/2 was equal to 64 ns and τ = 250 ns. Registration of the EPR spectra was performed by detecting the ESE integral intensity depending on the magnetic field value B_0_. The choice of the W-band was justified by the need to achieve a higher spectroscopic resolution (that allows identifying distinct EPR signals with close g-factors) and high sensitivity (to register weak EPR signals). The spectra were recorded both at room temperature (T = 297 K) and at lower temperature (T = 200 K). Stable paramagnetic centers were formed under X-ray irradiation of the synthesized powders using the URS-55 source (G = 55 kV, I = 16 mA, W-anticathode) at room temperature for 30 min with an estimated dose of 5 kGy and by illuminating the samples in the EPR cavity via laser sources with different wavelengths (λ) from 266 nm up to 355 nm.

The most abundant metal impurities in CaP detected with the EPR method are related to manganese, chromium, iron and copper. Low-temperature measurements and the use of high-frequency methods allowed us to increase the sensitivity of EPR. Details of the EPR measurements, including electronic relaxation times for HA, are given in ref. [43]. Simulations of the EPR spectra were performed by means of the EasySpin package [44].

### 2.3. Nuclear Magnetic Resonance

For all the samples, the MAS-NMR spectra, the times of spin–lattice and spin–spin relaxation on phosphorus (^31^P) nuclei and hydrogen (^1^H) protons were measured. For measurements, an AVANCE400WB NMR spectrometer (Karlsruhe, Germany) with a MAS 4BL CP BB DVT sensor was employed. The resonant frequency on protons was about 400.27 MHz, and on ^31^P nuclei, it was 162.034 MHz. The duration of a π/2 pulse on protons was 2.5 µs (output power of 94 W), and on 31P nuclei, it was 3.4 µs at a power of 50 W. The standard pulse programs, such as onepulseq, hpdec, cp, inversion-recovery and cpmg, were used to obtain NMR spectra and relaxation on ^1^H, ^31^P and ^13^C. The spectrum width for all measurements was 100 kHz. Powder samples were densely packed in a 4 mm zirconium oxide rotor and spun up to a rotation frequency of 7 kHz. The measurements were carried out at 295 K. The chemical phenomena related to the NMR signals were calibrated by measuring the signal of water (δ = 4.67 ppm) for protons and 85% phosphoric acid for phosphorus (δ = 0 ppm).

## 3. Results

### 3.1. SEM

**Figure 3:**
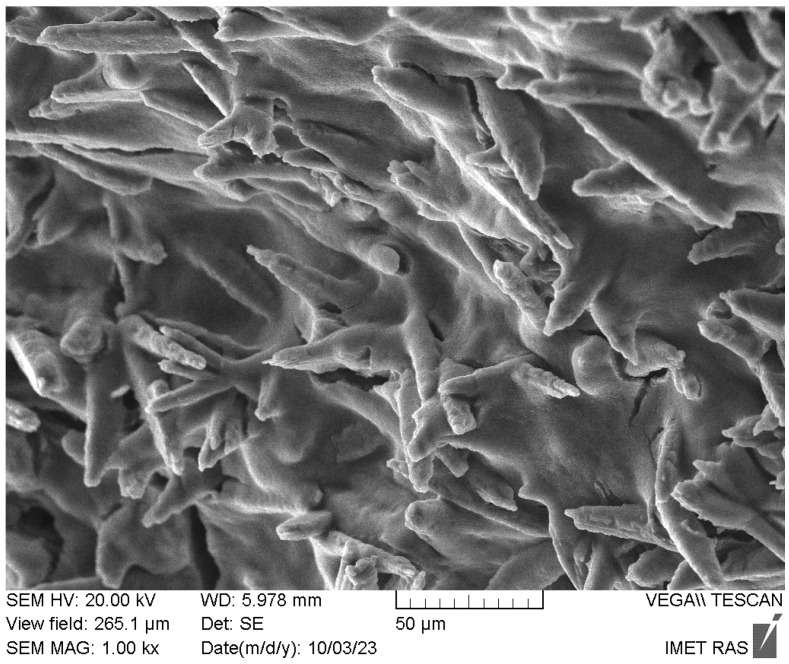
SEM image of the PVP-HA composite.

As it follows from the SEM experiments (Figure 3), the microstructure of the PVP-HA composite is represented by rod-shaped particles about 30 µm long, coated with polymer. It resembles the results reported in ref. [18]. The polymer facilitates the formation of the needle-like HA aggregates, whereas, in the absence of PVP, it is difficult to obtain a rod-like structure. This can be attributed to the Ostwald ripening process occurring in the solution and leading to a typical oriented attachment process, especially in the presence of PVP.

### 3.2. EPR

**Figure 4:**
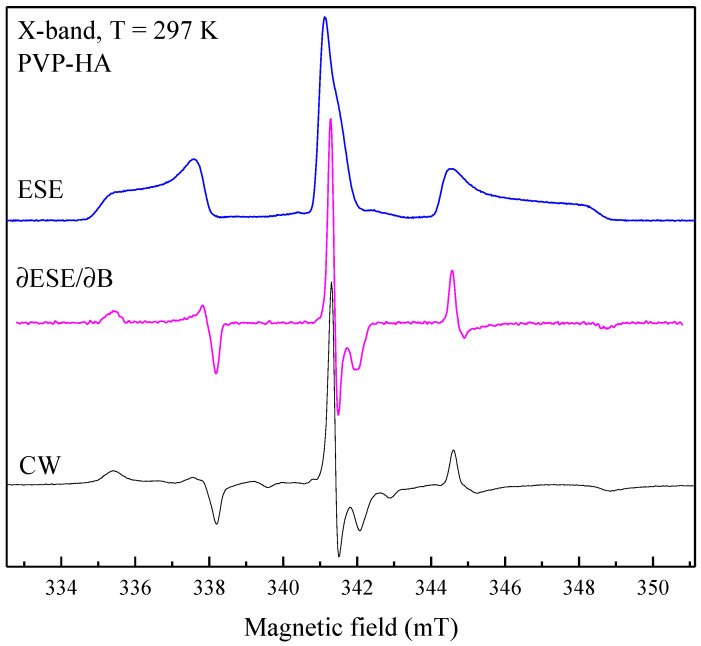
Comparison of EPR spectra of PVP-HA sample detected via ESE (blue upper curve) and cw (black lower curve) after X-ray irradiation in the X-band at T = 297 K. Middle curve was obtained by differentiating the ESE EPR spectra.

**Figure 5:**
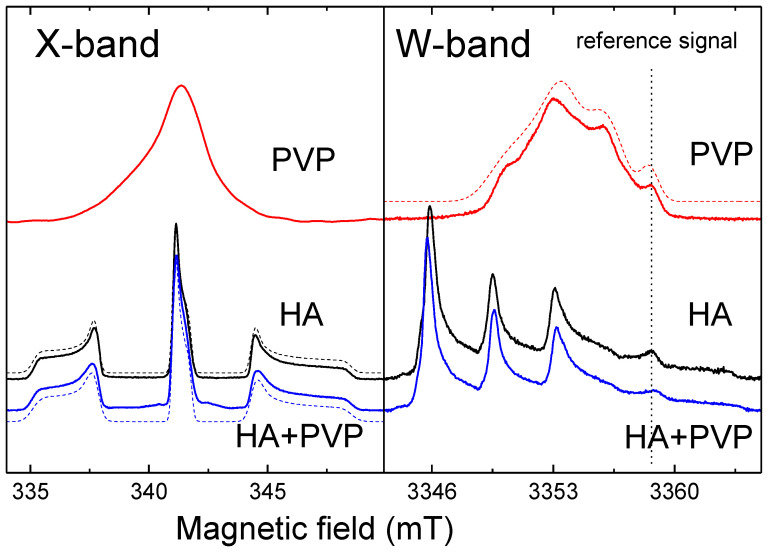
EPR spectra obtained by detecting the ESE of samples after X-ray irradiation: X-band in the the left panel, W-band in the right panel. The red lines show the spectra of PVP, the black lines the spectra of HA, and the blue lines the spectra of PVP-HA. The dotted lines show the approximations of the EPR spectra. The approximation parameters are given in Table 1.

**Figure 6:**
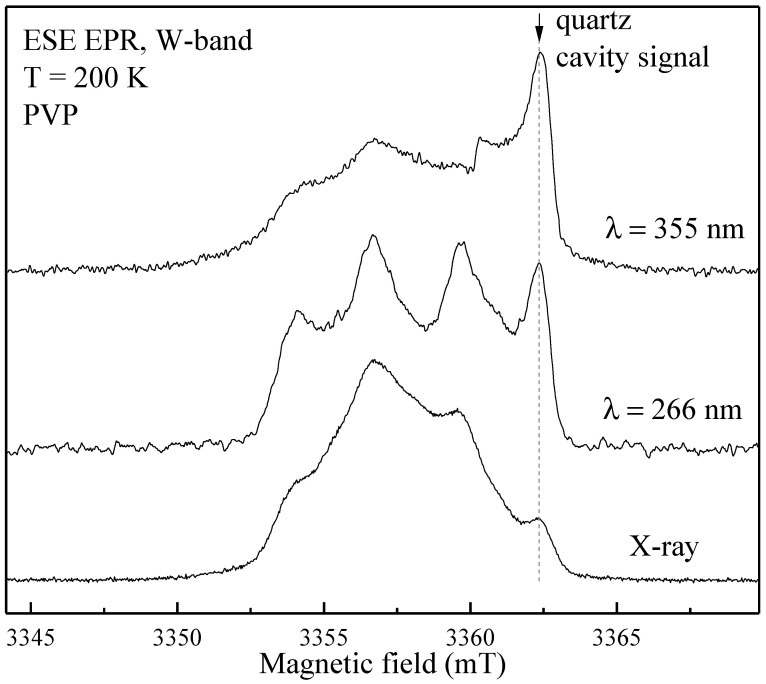
Photo-induced (using lasers with the wavelengths of 355 and 266 nm) and X-ray induced ESE EPR spectra at T = 200 K of PVP in the W-band. The dotted vertical line shows the signal from the quartz tube of the sample holder.

For HA, PVP and PVP-HA, the EPR signal was not observed due to the absence of paramagnetic centers in the structure of the materials. It additionally proved the purity of the initial materials and the reaction product. After the X-ray irradiation of HA powders in the X-band, the EPR spectrum containing three lines was registered (Figure 4). The spectroscopic parameters of the EPR spectrum allowed us to refer the detected signal to the well-studied NO_3_^2^ stable species in HA [45,46,47] (see Table 1), due to the hyperfine interaction (A) with the ^14^N nuclei (I = 1). The PVP samples were also studied after X-ray irradiation (Figure 5, red lines) and photo-induced EPR at low temperatures (Figure 5), since it is known that exposure to the visible light does not lead to the formation of stable radicals in HA [43], while it does in PVP polymer chains [48]. In the W-band, due to the higher spectral resolution, a three-line pattern of PVP was observed (Figure 6). It accounted for the localization of an unpaired electron on the PVP nitrogen atom (Figure 2) with a constant A_||_ = 106 ± 10 MHz. In the X-band, the three-line pattern for PVP is not resolved.

As can be seen from Figure 5 (blue lines), the EPR spectrum of the PVP-HA contains only the NO_3_^2^ radicals from HA, and the component from PVP was not observed. It can be supposed that in the PVP-HA mixture, the radiation-induced centers in PVP have a competitive electron trap channel, which is possible only in the presence of a chemical bond between the components. In ref. [49], we also showed that for the PVP-HA samples obtained ex situ, the values of the A-components and their distribution for the electron-^14^N interaction grew with the increase in the amount of PVP. A change in the crystal structure of HA in the reaction with PVP is unlikely. Therefore, we can assume that during the formation of chemical bonds, the PVP molecules create a positively charged layer around the HA particles, which increases the electron density in the near-surface layer of the HA particle.

Electronic spin–lattice relaxation measurements did not reveal an influence of PVP on the relaxation parameters of the radiation-induced NO_3_^2−^ radicals from HA, as is demonstrated in Table 2 for the X-band experiments. This correlates with the tiny influence of PVP on the EPR spectra of HA, as described above.

### 3.3. NMR

**Figure 7:**
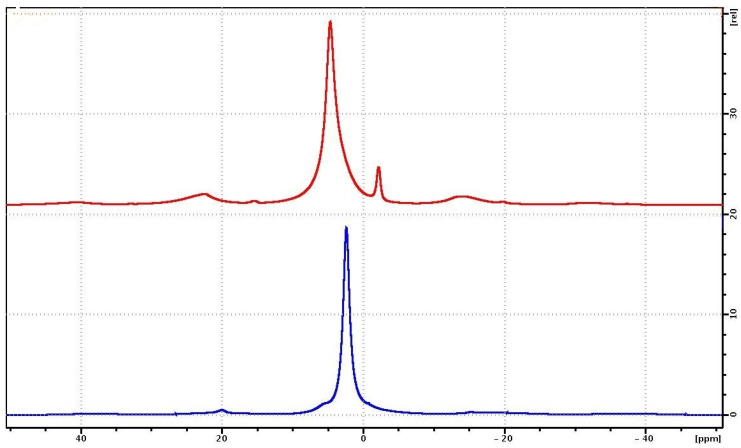
^1^H NMR spectra of PVP (blue curve) and PVP-HA (red curve) samples.

The ^1^H NMR spectra for PVP and PVP-HA are shown in Figure 7. The ^1^H MAS-NMR spectrum of the PVP consists of one unresolved NMR signal at 2.4 ppm, associated with the CH_2_ groups of the pyridine ring (Figure 2) and the polymer chain. The CH groups of the polymer chain contribute to the signal at about 5 ppm, while the CH_3_ end groups give a small contribution to the region of 0.5–1.5 ppm. On the ^1^H NMR spectrum in Figure 7, the contributions of these chemical groups are seen as small shoulders to the right and left of the main NMR signal.

The ^1^H NMR spectrum of a PVP-HA sample consists of two ^1^H NMR signals at 4.7 ppm and −2.15 ppm [50,51]. The integrated intensity of the NMR signal at 4.7 ppm is significantly higher compared to the intensity of the NMR signal at −2.15 ppm. The ^1^H NMR signal at 4.7 ppm for the PVP-HA is associated with «free» water adsorbed on the surface of HA, while the 1H NMR signal at −2.15 ppm is associated with hydroxyls present in the HA structure. The shape of the NMR signal at 4.7 ppm is asymmetric due to the superposition of the proton signals of water and polymer.

**Figure 8:**
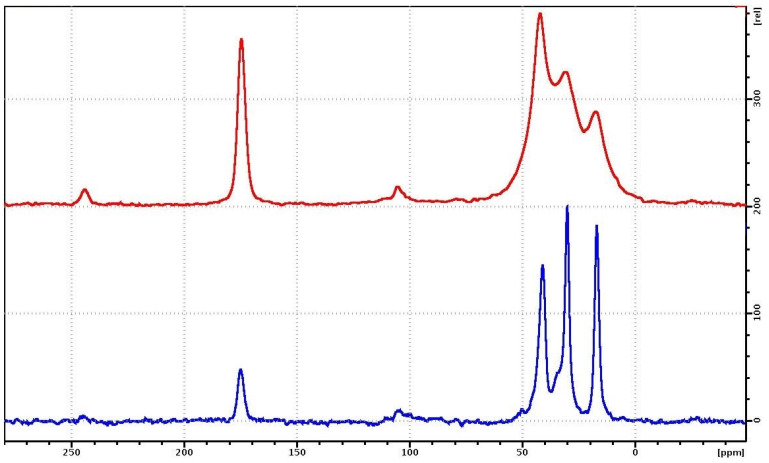
MAS-NMR spectra of ^13^C{^1^H} cp (red) and hpdec (blue) of the PVP. The rotation frequency of the sample is 7 kHz.

The ^13^C{^1^H} MAS-NMR spectra of the PVP on ^13^C during proton decoupling, obtained using the pulse program cp (red) and hpdec (blue), respectively, are shown in Figure 8. The MAS-NMR ^13^C{^1^H} spectrum of PVP-HA has the same form as in Figure 7, only the signal-to-noise ratio is several times lower. The NMR signals of the aliphatic region for cross-polarization have lower resolution than those present the ^13^C NMR spectrum with proton suppression.

The data obtained for the spin–lattice relaxation rates on protons for the two samples (PVP and PVP-HA) are shown in Table 3. The table lists the chemical shifts of the signals for which the spin–lattice relaxation rates were measured.

**Figure 9:**
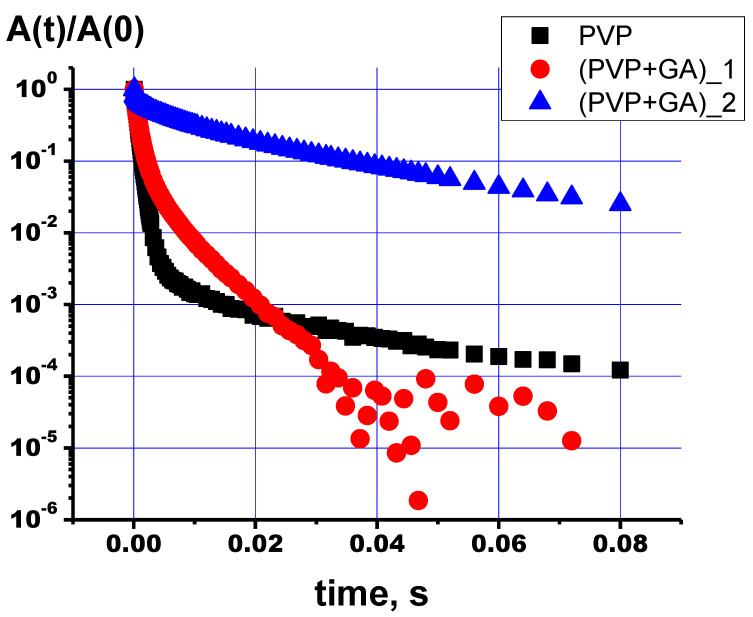
Relaxation decay curves of protons of NMR signals for PVP (black squares) and PVP-HA (red circles for protons with chemical shift 4.7 ppm and blue triangles for the signal at −2.15 ppm).

The relaxation decays obtained via the Carr–Purcell–Meiboom–Gill (cpmg) sequence for the NMR signals of the chemical shift regions, according to Table 3, are presented in Figure 9. As can be observed, the proton relaxation decays for the NMR signals from the PVP and PVP-HA differ from each other and can be described by exponent approximation. The decay curves of the transverse magnetization of protons in the PVP and PVP-HA samples were approximated by the following formula:

(2) $$A\left(t\right)=A\left(0\right)\cdot \sum_{i=1}^{n}p_{i}\cdot \exp\left(-\frac{t}{T_{2\left(i\right)}}\right),$$

where *A*(0) is the initial amplitude of the NMR signal and T_2(i)_ is the spin–spin relaxation times of components with populations *p_i_*. The values of the spin–spin relaxation times and their populations calculated from the relaxation decays are given in Table 4.

It is known that multicomponent relaxation can manifest itself as a result of different types of molecular motion or different surroundings of the nuclei. We assume that the presence of three transverse relaxation components for protons is due to the different positions of the OH groups in the PVP-HA composite. So, hydroxyls, included in the structure of HA (δ = −2.15 ppm), should be characterized by the longest time of transverse (spin–spin) magnetization. In the environment of the HA crystal defects, hydroxyls should be described by high rates of nuclear spin–spin relaxation. The same approach applies to water localized on the HA surface. 

**Figure 10:**
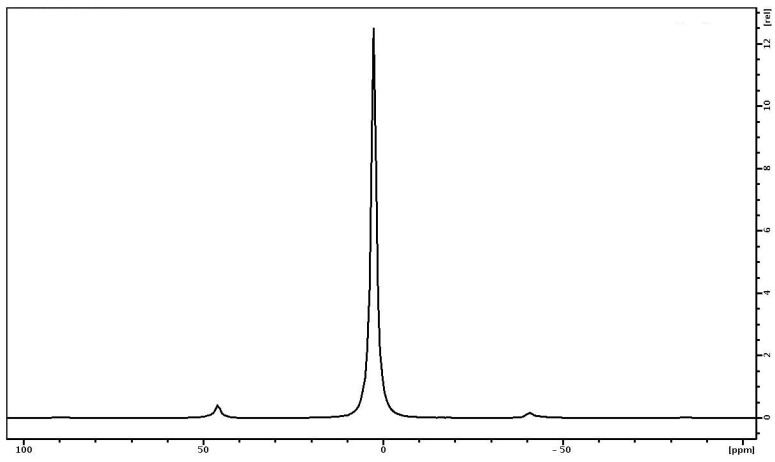
^31^P NMR spectrum of PVP-HA. The sample rotation frequency is 7 kHz.

The ^31^P NMR spectrum of the PVP-HA sample is shown in Figure 10. It is a single line with a width of about 290 Hz with an isotropic chemical shift of 3 ppm. The shape of the spectrum indicates that all the phosphorus nuclei in HA are in the same chemical environment. It should be noted that the stationary ^31^P NMR spectrum of the PVP-HA sample (without rotation) has the same chemical shift and a linewidth of 3000 Hz.

**Figure 11:**
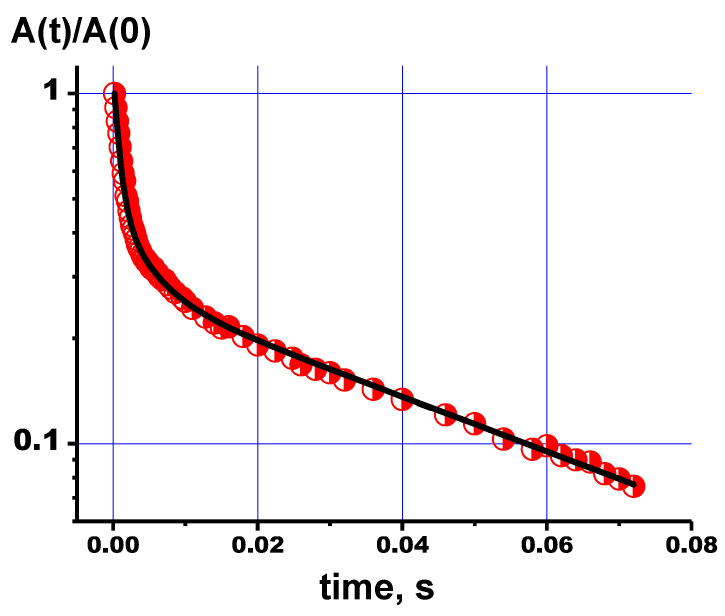
Relaxation decay of the ^31^P NMR signal for the PVP-HA. White/red circles are the experimental data. Black line is the approximation according to Equation (2) with the parameters listed in Table 5.

The spin–lattice relaxation time for ^31^P was measured as T_1_ = 238 ± 21 s. The relaxation decay of the ^31^P NMR signal for the PVP-HA and the fitting curve based on the relaxation decay decomposition parameters, according to Equation (2), are shown in Figure 11.

The values of spin–spin relaxation times and their populations obtained from the relaxation decay for the line in the ^31^P NMR spectrum of the PVP-HA are listed in Table 5. The presence of three transverse relaxation components both for phosphorus atoms and protons is probably due to the presence of crystal lattice defects in the HA.

**Table 5:** The values of ^31^P spin–spin (transverse) relaxation times of components with populations *p_i_*, according to Equation (2).

Chemical Shifts of NMR Signals (ppm)	*p*_1_	T_2(1),_ ms	*p*_2_	T_2(2),_ ms	*p*_3_	T_2(3),_ ms
PVP-HA						
3	0.244	55.5 ± 0.5	0.189	4.2 ± 0.3	0.567	0.85 ± 0.05

## 4. Conclusions

1. Using two microwave frequencies, the EPR parameters for the light- and radiation-induced paramagnetic centers in the PVP, HA and PVP-HA (such as components of g-factors and hyperfine constants A between electrons and ^14^N nuclei) were defined with high accuracy (Table 1). This shows the possibility of using EPR for the qualitative (synthesis, presence of impurities) and quantitative (concentration of impurities and defects) control of the initial materials (PVP, HA) and the final composite PVP-HA product.
2. In the PVP-HA composite, the EPR spectra and electronic relaxation times of the radiation-induced paramagnetic centers were very close to those in HA. In the PVP-HA, the distribution of A components for the electron–^14^N interaction for NO_3_^2−^ radicals was larger than for the pure HA (Table 1). The PVP-HA composites did not contain light-induced radicals characteristic of PVP. This can be ascribed to the re-distribution of the electrical charges between PVP and HA. These results can be used for the control of the quality and success of the in situ synthesis of PVP-HA composites using the EPR techniques.
3. In the ^1^H MAS NMR spectrum of the PVP-HA, the presence of two signals at 4.7 ppm and −2.15 ppm were attributed to “free” water and hydroxyl groups, and a single line attributed to ^31^P was registered.
4. The NMR relaxation measurements for ^1^H and ^31^P showed that the relaxation decays are multicomponent processes that can be described by three components of the transverse relaxation times. Multicomponent relaxation decay can be ascribed to the presence of defects in the HA lattice. The obtained data can serve as a basis for future NMR applications in clinical MRI for the evaluation of skeleton quality.
5. The obtained results demonstrate that the applied magnetic resonance techniques can be used for the quality control of synthesis products and, potentially, to follow the processes of the samples’ treatment, resorption and degradation [36,37].

## Figures and Tables

**Table 1:** Spectroscopic parameters of the observed EPR spectra obtained from their simulations in the X- and W-bands.

Sample	g_⊥_	g_||_	A_⫠_ (MHz)	A_||_ (MHz)	ΔA_⫠_ (MHz)	ΔA_||_ (MHz)
PVP	2.0022	2.0026	38 ± 8	106 ± 10	-	-
HA	2.0011	2.0052	92.4 ± 0.5	186 ± 1	7 ± 1	12 ± 1
PVP-HA	2.0011	2.0051	93.5 ± 0.5	190 ± 2	13 ± 1	18 ± 1

**Table 2:** Spin–lattice (T_1e_) and spin–spin (T_2e_) relaxation times of NO_3_^2−^ radicals measured in the X-band (v = 9.6 GHz) at the central peak (transition) of the hyperfine structure of the nitrogen radical at B_0_ = 341.2 mT (corresponds to g_⊥_).

	T_1e_ (μs)	T_2_ (μs)
HA	28.5	3
PVP-HA	27.6	3.2

**Table 3:** The values of ^1^H spin–lattice relaxation rates for PVP and PVP-HA.

Sample	2.4 ppm	4.7 ppm	−2.15 ppm
PVP	0.67 ± 0.02 s^−1^	-	-
PVP-HA	-	0.49 ± 0.02 s^−1^	0.55 ± 0.02 s^−1^

**Table 4:** The values of ^1^H spin–spin (transverse) relaxation times of components with populations *p_i_*, according to Equation (2).

Chemical Shifts of NMR Signals (ppm)	*p*_1_	T_2(1),_ ms	*p*_2_	T_2(2),_ ms	*p*_3_	T_2(3),_ ms
PVP						
2.4	0.0015	29.6 ± 0.5	0.0067	3.6 ± 0.1	0.992	0.59 ± 0.02
PVP-HA						
4.7	0.04	5.42 ± 0.2	0.18	1.67 ± 0.05	0.78	0.58 ± 0.02
−2.15	0.346	29.2 ± 0.5	0.33	6.02 ± 0.05	0.334	0.46 ± 0.03

## Data Availability

The experimental data on the results reported in this manuscript are available upon an official request to the corresponding author.
